# Borna disease virus (BDV) infection in psychiatric patients and healthy controls in Iran

**DOI:** 10.1186/1743-422X-11-161

**Published:** 2014-09-03

**Authors:** Elham Mazaheri-Tehrani, Nader Maghsoudi, Jamal Shams, Hamid Soori, Hasti Atashi, Fereshteh Motamedi, Liv Bode, Hanns Ludwig

**Affiliations:** Neuroscience Research Center, Shahid Beheshti University of Medical Sciences, P.O. Box 19615–1178, Tehran, Iran; Department of Diagnostic, Clinic and Public Health Medicine, University of Modena and Reggio Emilia, Modena, Italy; Behavioral Research Center, Shahid Beheshti University of Medical Sciences, Tehran, Iran; Safety Promotion and Injury Prevention Research Center, Shahid Beheshti University of Medical Sciences, Tehran, Iran; Bornavirus Working Group affiliated to the Free University of Berlin, Berlin, Germany

**Keywords:** Borna disease virus, Circulating immune complexes, Psychiatric disorders, Iranian patients/controls

## Abstract

**Background:**

Borna disease virus (BDV) is an evolutionary old RNA virus, which infects brain and blood cells of humans, their primate ancestors, and other mammals. Human infection has been correlated to mood disorders and schizophrenia, but the impact of BDV on mental-health still remains controversial due to poor methodological and cross-national comparability.

**Method:**

This first report from the Middle East aimed to determine BDV infection prevalence in Iranian acute psychiatric disorder patients and healthy controls through circulating immune complexes (CIC), antibodies (Ab) and antigen (pAg) in blood plasma using a standardized triple enzyme immune assay (EIA). Samples of 314 subjects (114 psychiatric cases, 69 blood donors, and 131 healthy controls) were assayed and data analyzed quantitatively and qualitatively.

**Results:**

CICs revealed a BDV prevalence of one third (29.5%) in healthy Iranian controls (27.5% controls; 33.3% blood donors). In psychiatric patients CIC prevalence was higher than in controls (40.4%) and significantly correlating with bipolar patients exhibiting overt clinical symptoms (p = 0.005, OR = 1.65). CIC values were significantly elevated in bipolar (p = 0.001) and major depressive disorder (p = 0.029) patients as compared to controls, and in females compared to males (p = 0.031).

**Conclusion:**

This study supports a similarly high prevalence of subclinical human BDV infections in Iran as reported for central Europe, and provides again an indication for the correlation of BDV infection and mood disorders. Further studies should address the morbidity risk for healthy carriers and those with elevated CIC levels, along with gender disparities.

## Background

*Borna disease virus* (BDV) holds unique features in terms of its cell biology, molecular properties, preference to old brain areas, broad host spectrum [1], and unusual biological age, dating back to more than 40 million years [2, 3]. The outstanding molecular biology of the virus, and its single

stranded RNA genome leading to the classification [4] of an own family, *Bornaviridae* (order *Mononegavirales),* has been comprehensively reviewed [5]. BDV had first been recognized as an often deadly pathogen of horses and sheep [1, 6] with a wide spectrum in other domestic and farm animals. However, BDV’s non-cytolytic properties, low replication while over-expressing two major proteins, and evidence of modulating neurotransmitter networks [7], pointed to a long-term adaption toward moderate pathogenicity and persistency [1].

Human infection and its putative link to mental disorders, first suggested after detection of antibodies [8], became a key issue inspiring research groups around the globe. After nucleic acid and antigen could be demonstrated in white blood cells of psychiatrically diseased patients [9], such a link was further strengthened by the finding of specific RNA sequences in post mortem brains of psychiatric patients [10] and limbic structures from old people [11].

The impact of human infection was significantly supported by the isolation and sequence characterization of human viruses from psychiatric patients’ blood cells and brain [12–14], and the recent correlation of neurological symptoms in humans with BDV infection [15]. The latest discovery of functional endogenous virus gene pieces integrated in the human and primate ancestor germ lines strongly argued in favor of a long-term co-evolution of virus and hosts [2, 3, 16]. However, a role of BDV, whatsoever, in human mental-health remained controversial, despite of predominantly supportive reports [17–24]. This is mainly due to a great variation in prevalence results largely caused by methodological disparities, due to different antibody and/or RNA techniques, affecting as well cross-national comparability. In contrast, BDV-specific circulating immune complexes, the most prevalent infection markers [25], have shown to be superior to antibody- or RNA- detection. Pilot prevalence studies could demonstrate that the BDV-CIC enzyme immune assay (EIA) is an easy to perform and robust test format, suitable to conducting comparable surveys in the general population of different countries, as well as longitudinal follow-up studies of patients in clinical cohorts [26–31]. Circulating immune complexes are the result of periods of antigenemia over-expressing N- and P-proteins, and antibody induction in the host, reflecting recent and current virus activity. Evidence for a contribution of BDV infection to disease symptoms has recently been reviewed [32].

This is the first report from the Middle East, addressing the prevalence of BDV in the human population in Iran. The virus in horses has previously been reported by antibody studies [33]. Here we explore the prevalence of BDV markers among Iranian mentally diseased patients, healthy controls, and blood donors.

## Method

### Individual subjects

Three hundred and fourteen Iranian subjects, including 114 psychiatric patients, 131 sex and age matched healthy controls, and 69 blood donors were included in this study. The association between BDV infection markers in blood plasma and five DSM IV- categorized psychiatric diseases, as well as gender and age of the individuals were analyzed.

Basic data are given in Table 1. One hundred and fourteen acute psychiatric patients, who had been admitted to local departments of psychiatry in Tehran, were included. All patients met the *Diagnostic and Statistical Manual of Mental Disorders IV* (DSM-IV) -criteria on the basis of interviews and medical records. They could be divided into five main groups and different DSM IV codes, including 64 bipolar disorder (BD)-, 12 major depressive disorder (MDD)-, 18 schizophrenia-, 15 schizoaffective- and 5 obsessive compulsive disorder (OCD) patients (Table 2). Additionally, 69 blood donors and 131 sex- and age matched, mentally healthy subjects (based on the supervision of the psychiatrists) were included and regarded as controls. All individuals were negative for Hepatitis B- and C-viruses, as well as HIV. The study was approved by the Ethic Committee of the Neuroscience Research Center at Shahid Beheshti University of Medical Sciences, and all patients -or an authorized representative- gave their written informed consent for participation. Blood samples of all individuals were collected prior to any medical treatment and plasma or sera were kept at -20°C.

**Table 1:** **Basic data on the population**

Groups	N	Female/Male	Mean age + SE	Min-Max
**Controls**	131	83/48	41.08 + 1.009	18-69
**Blood donors**	69	6/63	29.93 + 1.296	19-58
**Mental patients**	114	52/62	37.42 + 1.103	17-62
BD*	64	32/32	36.20 + 1.477	17-62
MDD**	12	7/5	43.42 + 3.450	21-57
Schizophrenia	18	3/15	34.56 + 2.689	20-53
Schizoaffective	15	5/10	38.33 + 2.863	22-57
OCD***	5	5/0	46.20 + 3.967	37-56
**Summary**	314	141/173	37.30 + 0.688	17-69

*Bipolar disorder.

**Major depressive disorder.

***Obsessive compulsive disorder.

**Table 2:** **DSM IV codes, numbers and symptoms of psychiatric patients**

Code (N)	Symptoms
**BD (N=64)**	
296.02 (2)	Single manic episode, moderate
296.03 (4)	Single manic episode, severe without psychotic features
296.04 (6)	Single manic episode, severe with psychotic features
296.42 (1)	Most recent episode manic, moderate
296.43 (5)	Most recent episode manic, severe without psychotic features
296.44 (20)	Most recent episode manic, severe with psychotic features
296.52 (2)	Most recent episode depressed, moderate
296.53 (6)	Most recent episode depressed, severe without psychotic features
296.54 (2)	Most recent episode depressed, severe with psychotic features
296.62 (2)	Most recent episode mixed, moderate
296.63 (11)	Most recent episode mixed, severe without psychotic features
296.64 (3)	Most recent episode mixed, severe with psychotic features
**MDD (12)**	
296.22 (2)	Recurrent, moderate
296.23 (1)	Recurrent, severe without psychotic features
296.24 (2)	Recurrent, severe with psychotic features
296.32 (3)	Single episode, moderate
296.33 (2)	Single episode, severe without psychotic features
296.34 (2)	Single episode, severe with psychotic features
**Schizophrenia (18)**	
295.01 (3)	Disorganized type
295.03 (9)	Paranoid type
295.09 (6)	Undifferentiated type
**Schizoaffective (15)**	
295.07 (15)	Schizoaffective disorder
**OCD (5)**	
300.03 (5)	Obsessive compulsive disorder

### Enzyme immune assays (EIAs)

The BDV infection markers, circulating immune complexes (CICs), virus antigens (N- and P- protein, N/P-complexes; abbreviated Ag), and antibodies (Ab), were assayed using the triple enzyme immune assay (EIA) system, as described [25]. According to the double- sandwich format, two BDV-specific monoclonal antibodies (mAbs), anti-N mAb (W_1_) and anti-P mAb (Kfu_2_), were used to bind any BDV-N- and P-protein or N/P -heterodimers in plasma, either circulating antigen bound to virus- specific host antibodies (CIC- EIA) or free antigen (pAg-EIA). CICs were visualized through alkaline phosphatase (AP)-coupled anti-human IgG and substrate, whereas the Ag-EIA needs a BDV-specific detecting antibody (rabbit hyper-immune serum) followed by AP phosphatase coupled anti-rabbit IgG and substrate. The specificity and sensitivity of the BDV mAbs have been further characterized [34]. In particular, epitope mapping has revealed that both these mAbs are binding to powerful conformational epitopes on either protein, which are formed through 5 binding sites in case of the anti-N mAb (W_1_) and 3 binding sites in case of the anti-P mAb (Kfu_2_). None of the W1 binding sites are overlapping with P-protein binding domains on the N-protein, confirming that commonly occurring N/P heterodimers are recognized by W_1,_ as well. In addition, none of either W_1_- and Kfu_2_- binding sites are overlapping with functionally important sites on N and P-protein, like NLS (nuclear localization signal) and NES (nuclear export signal). The extraordinarily high binding capacities of these mAbs to native N and P proteins have been determined through affinity-chromatography methods using N and P protein from the brain of a horse with Borna disease, resulting in dissociation constants (K_D_) of 2.31 × 10^-9^ for W1 and 3.33 × 10^-9^ for Kfu2. Like for other antigen assays, recombinant proteins have been used to determine sensitivity and further confirm specificity. The detection limit of 1.5-3 ng/ml of purified recombinant N-protein (rN) has been determined for W_1_ mAb. Diluting of rN in CIC-, Ag- and Ab- EIA-negative serum did not make any difference, confirming specificity. Additionally, N-protein could be demonstrated in the immune precipitate (IP using W_1_) of a strongly antigen positive patient’s plasma by western blot, whereas the IP of an antigen-negative plasma showed nothing but the heavy and light chain of the mAb [34]. Furthermore, using recombinant P-protein, either the non-phosphorylated or phosphorylated form, revealed that mAb Kfu_2_ only detects the activated phosphorylated form. Regarding the antibody assay we followed the exact protocol given earlier [25]. All three assays use the basic coating of antibody-stabilized monoclonal antibodies (W_1_ and Kfu_2_) as a standard immune module [34].

According to the primary experimental setting, a standardized cut off value has been specified as a mean of negative values plus 2 standard deviations, regularly reaching an extinction of < = 0.1 which separates negative and positive scores. The initial dilutions of the samples were 1:20, 1:2, and 1:100 to allow the same cut off value for testing CICs, free Ag, and Ab, respectively [25]. Results were visualized through alkaline phosphatase conjugated antibodies and a colorimetric substrate, absorbance measured in a multichannel photometer (405 nm), and values imported to statistical software [25].

Repetition of one third of the sample collection was performed and essentially gave the same results.

### Statistical analysis

All data of the patients and controls were submitted to parametric and non-parametric statistical analyses. A comparison of the groups was carried out using independent T-Tests, ANOVA and Chi square tests. The prevalence of BDV infection markers was calculated as based on the cut off value of 0.1. Subjects were classified according to clinical diagnostic, gender and age as independent variables, as based on the CIC data measured.

The detailed evaluation of CIC tests were based on standardized scoring of the OD-values of >0.100- 0.300 to be +, >0.300- 0.600 to be ++, > 0.600 - 1.000 to be +++, and > 1.000 to be ++++ [25]. Prevalence and odds ratios (OR) were calculated. Chi square tests were used for an estimation of statistical differences between the groups. Furthermore, binary logistic regression for an estimation of an individual influence of three basic variables, namely age, gender and clinical diagnosis, on CIC titers was applied.

## Results

### Population characteristics

As shown in Table 1, efforts have been made to include gender and age matched control subjects, but comparability could finally not be achieved. The large disparity in both the female-to-male ratios and age of blood donors compared to patients considerably accounted for this limitation (gender: chi square = 7.758, p = 005; age: by t test, p = 0.015). As shown in Table 2, the majority of bipolar patients (BD) were either manic (59.4%) or in a mixed episode (25%), whereas only 15.6% experienced a recent depression. Of all patients, only 19.3% (10 BMD, 12 MDD patients out of 114) presented with a recent depressive episode.

### Circulating immune complexes

Based on CICs we found a mean prevalence of subclinical infection of 29.5% in the healthy Iranian controls, displaying a slightly higher prevalence in blood donors (33.3%) as compared to the healthy subject cohort (27.5%) for whom any mental illness has been excluded.

Gender and age had no significant influence on CIC prevalence, but psychiatric patients showed significant differences compared to the control group (p = 0.036), presenting with a mean CIC prevalence of 40.4%. Particularly, the patients with bipolar disorder were statistically significantly different with reference to CIC prevalence, OR and OR estimate (OR Est.) (p = 0.014). It is noteworthy that the CIC prevalence found in patients with mood disorders (BD, MDD, and schizoaffective disorders; N = 91) was doubling that of schizophrenia patients (44% vs. 22%), a difference which turned out to be statistically significant (p = 0.026), as well. The statistical evaluations are given in Table 3.

**Table 3:** **CIC results against three predictors: sex, age and diagnosis**

Predictors	N	Pos./Neg. (p %)	OR	OR Est.	CI (95%)
**Diagnoses:**					
Controls	131	36/95 (27.5%)	Ref	Ref	Ref
Blood donors	69	23/46 (33.3%)	1.21	1.029	0.483-2.193
Patients	114	46/68 (40.4%)	1.47	1.088	1.042-3.405
BD	64	29/35 (45.3%)	1.65	2.035	1.072-3.863
MDD	12	6/6 (50.0%)	1.82	2.750	0.820-9.222
Schizophrenia	18	4/14 (22.2%)	0.81	0.632	0.186-2.149
Schizoaffective	15	5/10 (33.3%)	1.21	1.254	0.392-4.014
OCD	5	2/3 (40%)	1.46	2.225	0.343-14.437
**Sex:**					
Male	173	57/116 (32.9%)	Ref	Ref	Ref
Female	141	48/93 (34.0%)	1.03	0.961	0.549-1.682
**Age group:**					
18-25	78	32/46 (41.0%)	1.948	2.962	0.850-10.325
26-35	66	24/42 (36.4%)	1.727	2.422	0.691-8.894
36-45	80	23/57 (28.8%)	1.365	1.597	0.469-5.440
46-55	71	22/49 (31.0%)	1.471	1.830	0.534-6.278
56-65	19	4/15 (21.1%)	Ref	Ref	Ref

### Free antibody and antigen

Based on the cut-off value of 0.1 [25] valid for all tests of the triple-EIA system to differentiate the negative from positive results, free Abs were measured in 7.8% and 16.7% of the bipolar (BMD) and schizophrenia patients, respectively, whereas the controls presented with 5.3%. Free Ag was present in 5.6% of the schizophrenic patients (1 out of 18), vs. 1 % in the controls (2 out of 200). Other patient groups were negative in both tests (for details see Table 4). The dynamic balance between CIC formation, antigens, and antibodies accounts for their relative amounts simultaneously present in a sample. The cross-sectional design of the study provides an infection profile only valid at a given time point, thereby limiting the explanatory power of triple-EIA results.

**Table 4:** **Prevalence of free antibodies and antigen**

	Free antibody			Free antigen (N-& P-protein)		
Groups	Pos./Neg.	Prevalence %	CI	Pos./Neg.	Prevalence %	CI
**Controls**	7/124	(5.3%)	1.5-9%	1/130	(0.7%)	0.7-2.3%
**Patients**	8/106	(7.0%)	2.3-11.7%	1/113	(0.9%)	0-2.6%
BD	5/59	7.8%	1.2-14.4%	0/64	0.0%	-
MDD	0/12	0.0%	-	0/12	0.0%	-
Schizophrenia	3/15	16.7%	0-33.9%	1/17	5.6%	0-16%
Schizoaffective	0/15	0.0%	-	0/15	0.0%	-
OCD	0/5	0.0%	-	0/5	0.0%	-
**Blood donors**	1/68	(1.4%)	0-4.2%	1/68	(1.4%)	0-4.2%
**Total**	16/298	(5.1%)	2.7-7.5%	3/311	(1%)	0-2%

### Additional data analysis

According to the cut off values, as shown in Table 5, dividing all data into a negative and positive group and performing only non-parametric analyses resulted in many data unavailable for statistical inference. Instead, we used quantitative CIC data from the EIA-reading (after subtraction of the OD values for blanks) for parametric statistical analyses.

**Figure 1:**
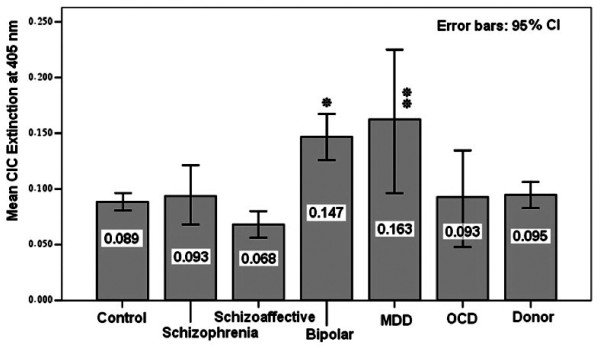
**Mean and 95% confidence intervals for CIC extinction in the investigated groups.** Lower and upper limits of 95% CI in groups including Control: 0.080-0.095, Schizophrenia: 0.068-0.121, Schizoaffective: 0.056-0.080, Bipolar: 0.125-0.167, MDD: 0.096-0.224, OCD: 0.047-0.134 and Donor: 0.083-0.105 based on Table 3. *Significant when compared to controls (p = 0.001, ANOVA). **Significant when compared to controls (p = 0.029, ANOVA). Extinction values refer to 1:20 dilution of plasma in the CIC-ELISA.

**Figure 2:**
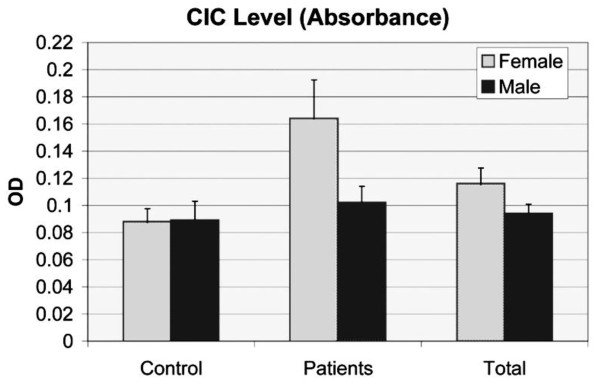
**Statistical differences between female and male samples in control and patient groups based on 95% CIC absorbance (p = 0.031).** 131 control samples (83 female and 48 male) and 114 patient samples (52 female and 62 male) were calculated by a parametric t-student test.

A noticeable increase in CIC levels of both, the bipolar disorder (0.147) and the major depressive disorder (0.163) groups became obvious, being statistically significant when compared to control subjects. The values for 95% CI of CICs are illustrated in Figure 1. The CIC levels within the total population tend to be elevated among females when compared to males (p = 0.089). Therefore, the influence of sex on CIC extinction values was also analyzed in these patient groups (Figure 2). A significant increase in CIC levels in female patients was recognized when compared to males (p = 0.031).

**Table 5:** **Distribution of categorized CIC results (neg., +, ++, +++) in subgroups**

Subgroups	Neg N (%)	+	++	+++	Total
**Controls**	95 (72.5%)	31 (23.7%)	5 (3.8%)	0	131
**Blood donors**	46 (66.7%)	21 (30.4%)	2 (2.9%)	0	69
Case	68 (59.6%)	36 (31.5%)	8 (7.0%)	2 (1.7%)	114
BD	35 (54.7%)	22 (34.4%)	6 (9.4%)	1 (1.60%)	64
MDD	6 (50.0%)	5 (41.7%)	0	1 (8.30%)	12
Schizophrenia	14 (77.8%)	2 (11.1%)	2 (11.1%)	0	18
Schizoaffective	10 (66.7%)	5 (33.3%)	0	0	15
OCD	3 (60.0%)	2 (40.0%)	0	0	5
**Sex**					
Male	116 (67.1%)	50 (28.9%)	7 (4.0%)	0	173
Female	93 (66.0%)	38 (27.0%)	8 (5.7%)	2 (1.4%)	141
**Age groups**					
18-25 ys	46 (59.0%)	26 (33.3%)	6 (7.7%)	0	78
26-35 ys	42 (63.6%)	20 (30.3%)	4 (6.1%)	0	66
36-45 ys	57 (71.3%)	20 (25.0%)	2 (2.5%)	1 (1.3%)	80
46-55 ys	49 (69.0%)	20 (28.2%)	2 (2.8%)	0	71
56-65 ys	15 (78.9%)	2 (10.5%)	1 (5.3%)	1 (5.3%)	78

OD absorbance is valued as (+): OD absorbance > 0.100 - 0.300, (++): OD absorbance > 0.300 - 0.600 and (+++): OD absorbance > 0.600.

## Discussion

This is the first study in Iranian people showing a fairly high prevalence of Bornavirus infection in healthy individuals including blood donors. The results meet reported data from Central Europe of about 30% based on the same infection marker (CIC). The study also supports previous findings that this neurotropic virus infection is more prevalent in psychiatric patients than in healthy donors. According to trends our results are supporting infection patterns in other countries, like Europe, the Americas and Asia which are based on specific antibody- and nucleic acid detection [9, 10, 15, 21, 35–42], despite of largely differing prevalence data. Based on measuring BDV released antigens or antigen-antibody complexes, like CICs [25, 27, 29, 30], our data showed a much better agreement.

Studies questioning and reporting the absence of BDV in both normal and psychiatrically diseased people remain inconclusive as long as no other cohorts have been investigated and no other methods have been applied. Among those are studies of Na et al. [42] and Hornig et al. [43].The latter group even neglected an own earlier positive study with contradictory results from the same country [10]. On the other hand, the existence of a human BDV strain has recently been independently proven by an *in vitro* study in brain cells. Only the human virus was able to reduce proliferation and enhance apoptosis but not the animal-derived laboratory strain of BDV [44].

Our study used an established triple EIA which had been successfully applied to monitor point- and longitudinal prevalence of BDV infection markers in patients [25, 26]. In our hands, these EIAs were found to be easy to handle and to provide robust and reproducible measurements. It is unfortunate that general acceptance is still pending. In this study, consecutive sampling of admitted patients was not possible. Although the data only refer to cross-sectional sample analysis, BDV markers were significantly more prevalent in Iranian patients with mental diseases than in control subjects. These findings were similar to data reported from Germany [25, 26], Italy [27] Australia [29], the CSSR [30], China (Xia Liu, Peng Xie, pers. communication), and Lithuania (Violeta Mockeliūnienė, Robertas Bunevicius, pers. communication) where the same test system had been applied.

The presence of CIC with or without antibodies indicates a chronic infection; the presence of Ag, with or without CICs at the same time, a currently active infection. The finding of free anti-BDV antibody alone (no antigen, no CICs) is thought to indicate previous exposure to the agent, but not a current active infection [34]. As shown in earlier reports CICs represent the major viral marker explaining the transient disappearance of antibodies and antigens in blood plasma between activated and dormant phases of virus infection, and by this providing also a clue for the true number of silently infected carriers in a healthy cohort or population [25, 26, 34].

Iranian psychiatric patients show a clearly elevated CIC sero-prevalence (40.4%) compared to healthy controls (27.5%). It is of special interest that 33.3% of samples from blood donations were silent virus carriers, a finding confirming Australian [29] and German pilot reports [17, 26], thus being quite in contrast to an earlier report [45]. Transfusion issues relating to BDV infection are still awaiting further clarification [46].

BD, MDD and OCD patients presented with infection rates of 45.3%, 50.0% and 40.0%, respectively. However, significance levels were only reached in BD patients. This might be due to the small sample size, but in parametric data analysis, comparing OD values of absorbance (extinction), high levels of CICs in sera from BD and MDD patients were also significant.

In contrast to other reports [47, 48], we found a relatively high sero-prevalence of free Ab and Ag in schizophrenic patients (16.7% and 5.6%, respectively) which is consistent with a relatively low CIC sero-prevalence among those individuals (22.2%, see Table 2). In addition to schizophrenic patients, only BD patients showed free antibodies in their sera (7.8%). This implies that BDV antibodies are usually bound in immune complexes and are therefore becoming transiently absent in the blood stream.

It is of considerable interest that the CIC sero-prevalence adversely correlated with the corresponding age groups (linear regression done using age as continuous data, R = -0.116, p = 0.042), which means that the young patients had highest CIC values, although the age limit includes only adults 18 years and older. This leaves the question whether younger people are either more prone to BDV infection or their immune response is more prominent. It supports a recent finding that young children (from 4–6 months to 3 years of age) had even much higher infection rates, although this pilot study warrants further investigations [28, 34], In addition, it has to be further examined whether and to which extent vertical transmission of BDV in the pregnant horse [49], mouse [50] and human [28] contributes to higher infection rates at young age. In this regard, high prevalence of BDV in the normal population, lifelong persistence of the virus in infected subjects (patients or healthy people), and the so far undisclosed function of endogenized BDV genome stretches [2, 3, 16], might reflect further risk factors warranting urgent future investigations.

Interestingly, significant differences between female and male patients could be measured for the first time (Figure 2. middle, p = 0.031), showing a prevalence of CICs in 42.3% of females, and 38.7% in males. In favor of these findings, two female patients, belonging to the BD and MDD groups, had high CIC titers with levels above 0.6 (+++) (Table 5). The sero-prevalence among healthy controls, however, reached only 25.3% in females and 31.3% in males.

Such sex-related specific differences according to, titers and prevalence of an antibody response to foreign antigens, infectious agents, or even auto antigens are known from the literature [51–55]. Females usually exhibit a stronger humoral immune response, as especially known after vaccination and infection with microbial agents. In fact, estrogens exert stimulatory effects on B cell proliferation and serum IgG levels, whereas testosterone may suppress B cell function [56, 57].

In conclusion, Iranian people seem to fit into the pattern of BDV infections, so far reported worldwide [5]. Moreover, the study benefits from using prevalent infection markers and a highly specific and effective test system [26, 34]. The study confirms evidence for a high infection prevalence, similar to Central Europe, in one third of healthy Iranian subjects, contrasting elevated levels in patients with mood disorders. In view of millions of people worldwide suffering from depression and the huge related health care costs [58], this study points again to integrating BDV infection surveillance in psychiatric research [26] rather than to continue in underplaying its impact.
